# Computer-Aided Design/Computer-Aided Manufacturing (CAD/CAM)-Assisted Prosthetic Rehabilitation When Physiologic Anterior Guidance Cannot Be Re-established in a Skeletal Class III Patient: A Clinical Case Report

**DOI:** 10.7759/cureus.115473

**Published:** 2026-08-30

**Authors:** Karami Elmfeddal, Alaa Samadi, Amal El Yamani

**Affiliations:** 1 Prosthodontics, Faculty of Dental Medicine, Mohammed V University, Rabat, MAR

**Keywords:** anterior guidance, cad/cam, fixed prosthodontics, skeletal class iii, virtual articulator

## Abstract

Prosthetic rehabilitation of a nonfunctional anterior guidance that cannot be restored represents a major clinical challenge when skeletal discrepancies prevent the re-establishment of physiological guidance. This clinical case report describes a 44-year-old patient presenting with skeletal Class III mandibular prognathism associated with edge-to-edge and anterior crossbite occlusal relationships. The contribution of computer-aided design and computer-aided manufacturing (CAD/CAM) technology to the esthetic and functional management of this complex situation is illustrated.

Following periodontal therapy and endodontic retreatment, an occluso-prosthetic compromise was established based on the absence of anterior contacts in maximum intercuspation (MI), simultaneous bilateral posterior stability, and posterior group-function guidance. Virtual treatment planning, performed using a mathematical virtual articulator driven by a dual physical-digital mounting protocol, enabled accurate restoration design and facially guided esthetic integration. The treatment plan was clinically validated using CAD/CAM-made provisional restorations and worn for three months before the fabrication of the final prostheses. The follow-up period was two years.

This case demonstrates that managing non-functional guidance using CAD/CAM technology can be clinically feasible when anatomical limitations necessitate a therapeutic compromise.

## Introduction

Anterior guidance corresponds to the set of occlusal contacts established between the maxillary and mandibular anterior teeth. It plays a key role in controlling mandibular movements and providing biomechanical protection to the teeth against excessive functional loads [1].

Anterior guidance is determined by guidance angles that depend on the morphology of the palatal surfaces of the maxillary incisors and canines. These angles are characterized by the incisal overjet, which determines the inter-arch relationships in the horizontal plane, and the incisal overbite, which determines the vertical relationships [2,3].

The biomechanical description during the occlusal examination allows differentiation between functional and non-functional anterior guidance. The former is characterized by well-distributed anterior contacts during propulsion and lateral movements, posterior disocclusion, and unrestricted mandibular movements. The latter, on the other hand, is characterized by a greatly increased (excessive overjet) or non-existent (anterior open bite or reverse occlusion) range of motion preventing any anterior contact during mandibular functions; the guidance is then considered nonfunctional. In this situation, restoring anterior guidance becomes essential during prosthetic rehabilitation to improve aesthetic and functional integration [4].

However, in certain clinical situations, such as severe sagittal skeletal discrepancies (skeletal Class II or Class III) or vertical discrepancies such as hyperdivergence (the height of the lower facial third increased relative to the upper), reproduction of a physiological anterior guidance using prosthetic restorations may be impossible [5].

The clinical strategy in the face of this impossibility of restoring anterior guidance will be based on a therapeutic compromise founded on the functional integration of the prostheses within the adapted malocclusion, as well as a dento-facial aesthetic integration [6].

With the increasing development of digital systems, managing such complex situations is becoming increasingly easier. Computer-aided design/computer-aided manufacturing (CAD/CAM) now offers the possibility of rigorously rehabilitating anterior guides thanks to the precision of the acquisition, enabling virtual simulation of occlusion in both its static and kinetic aspects using virtual articulators [7]. Furthermore, it allows for optimal aesthetic integration by facilitating personalized virtual aesthetic planning and standardized manufacturing [8].

The purpose of this clinical report is to illustrate, through a well-documented clinical case, the contribution of CAD/CAM technology to the esthetic and functional management of a nonfunctional anterior guidance in which physiological guiding inclines could not be restored due to skeletal Class III mandibular prognathism. Particular emphasis is also placed on the continued importance of conventional techniques within the digital workflow.

## Case presentation

Clinical examination

**Figure 1:**
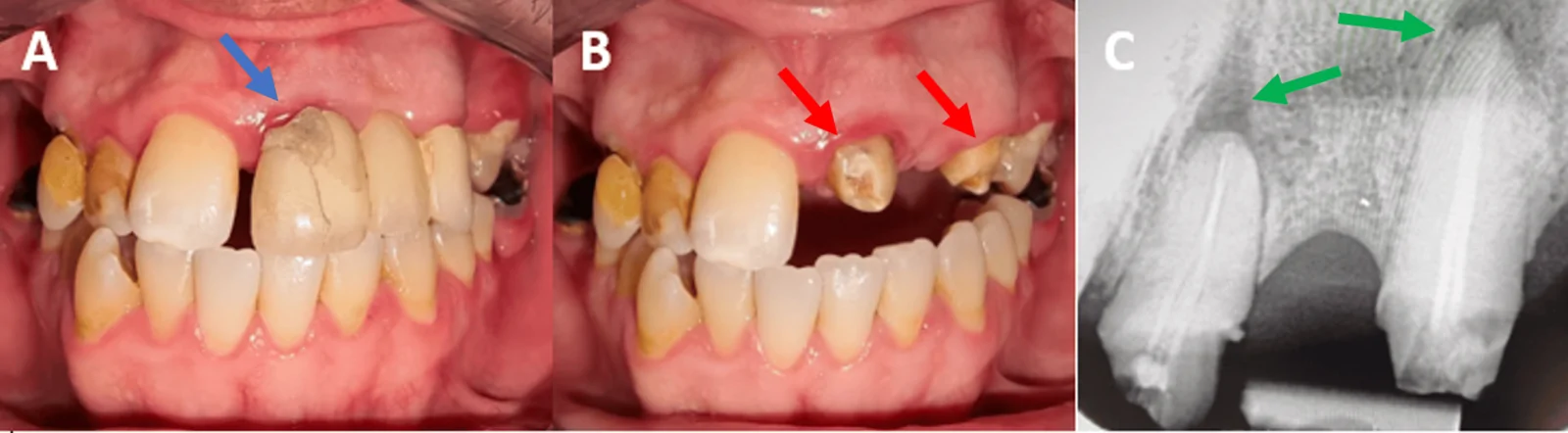
(A) Clinical view of the initial state. The blue arrow shows the fracture of the ceramic and the gingival bleeding at the level of the 21. (B) Removal of the bridge; the green arrows show the significant tissue damage of the abutment teeth 21 and 23. (C) Periapical radiograph; the blue arrows show the periapical reactions in relation to the defective quality of the endodontic treatments on teeth 21 and 23.

A 44-year-old patient consulted our fixed prosthodontics department following increased mobility of his bridge spanning teeth 21 to 23, which had been worn four years prior. He expressed significant concern about a ceramic fracture associated with bleeding and gingival pain over tooth 21 (Figure 1A, blue arrow). Emergency removal of the bridge revealed abutments with poor retention and significant tissue destruction (Figure 1B, red arrow). Periapical radiographs of the bridge abutments showed inadequate endodontic treatment associated with periapical reactions. No signs of tooth fracture were found (Figure 1C, green arrow)

**Figure 2:**
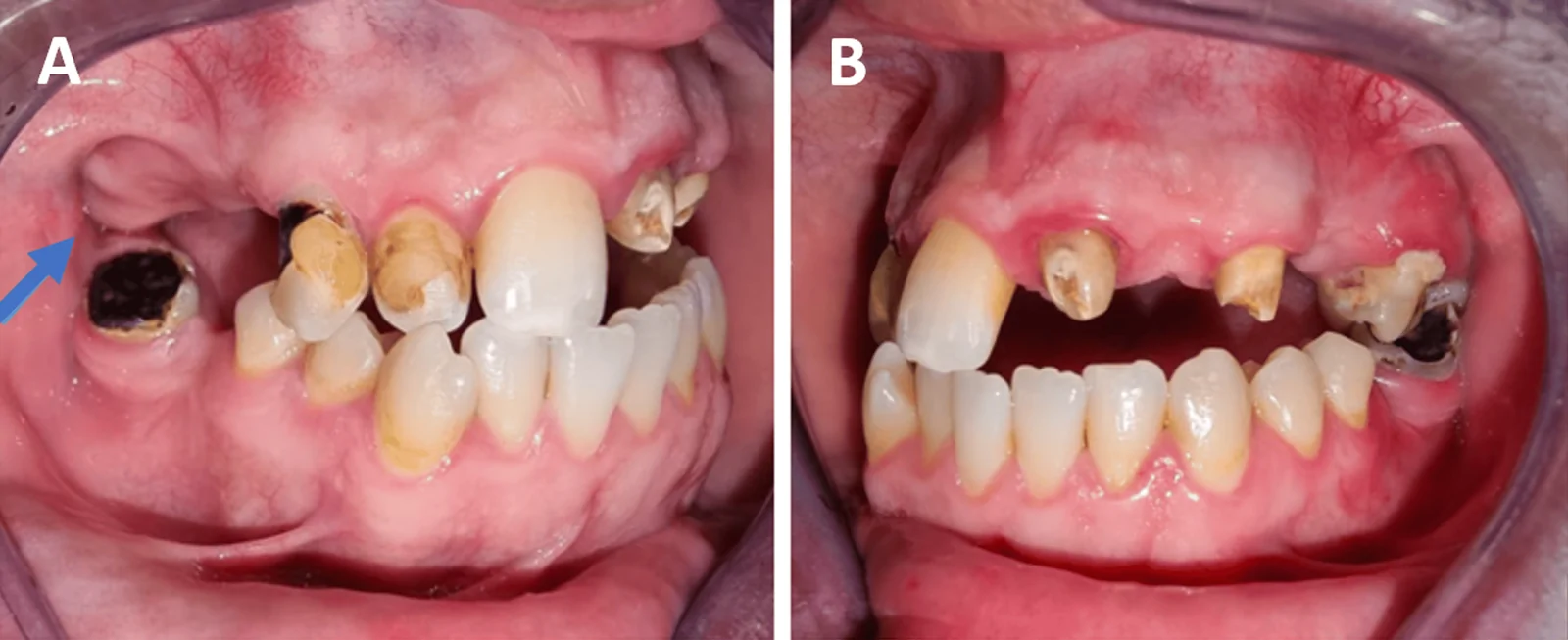
Right (A) and left (B) lateral intraoral views.

A full-mouth dental examination revealed defective composite restorations with recurrent caries on teeth 12 and 13 as well as extensive caries on tooth 47 (Figure 2A). On the left side we note the presence of coronal destruction due to caries on teeth 26 and 37 (Figure 2B). 

Regarding the periodontal examination, mechanical control of dental plaque was considered insufficient due to brushing being limited to a single daily occurrence. Probing revealed no periodontal pockets; sulcus depths ranged from one to three millimeters (Table 1). Furthermore, no pathological tooth mobility was observed. However, over 30% of the examined sites bled upon probing. Therefore, a diagnosis of generalized gingivitis was made without pathological involvement of the deep periodontal structures.

**Table 1:** Maxillary and mandibular periodontal probing depths PD: Probing depth

Anatomical site	Probing depth															
Maxillary teeth	18	17	16	15	14	13	12	11	21	22	23	24	25	26	27	28
Vestibular PD	-	-	-	-	-	2.2.2	2.1.3	2.2.3	3.3.3	-	3.2.1	-	-	3.2.1	1.2.2	-
Palatal PD	-	-	-	-	-	2.2.2	2.2.2	2.2.2	3.3.3	-	2.1.2	-	-	2.2.2	3.2.2	-
Mandibular teeth	48	47	46	45	44	43	42	41	31	32	33	34	35	36	37	38
Vestibular PD	-	1.3.3	-	1.1.1	1.1.1	2.2.3	3.3.3	2.2.2	3.2.3	3.1.3	1.3.2	2.2.2	1.1.1	-	1.1.2	-
Lingual PD	-	3.3.2	-	2.2.2	1.2.2	2.2.3	2.2.2	2.2.3	2.2.2	1.1.3	1.1.1	3.2.1	2.2.2	-	2.3.3	-

Occlusal analysis revealed a forward positioning of the mandibular arch responsible for edge-to-edge and anterior crossbite relationships between the anterior teeth. Furthermore, the loss of the anterior bridge was the cause of the lack of well-distributed anterior contacts during mandibular movements, resulting in non-functional anterior guidance. In the molar region, a significantly reduced prosthetic occlusal space was observed, associated with contact between the fibromucosal structures of the upper and lower right retromolar areas, clearly visible clinically (Figure 2, blue arrow).

**Figure 3:**
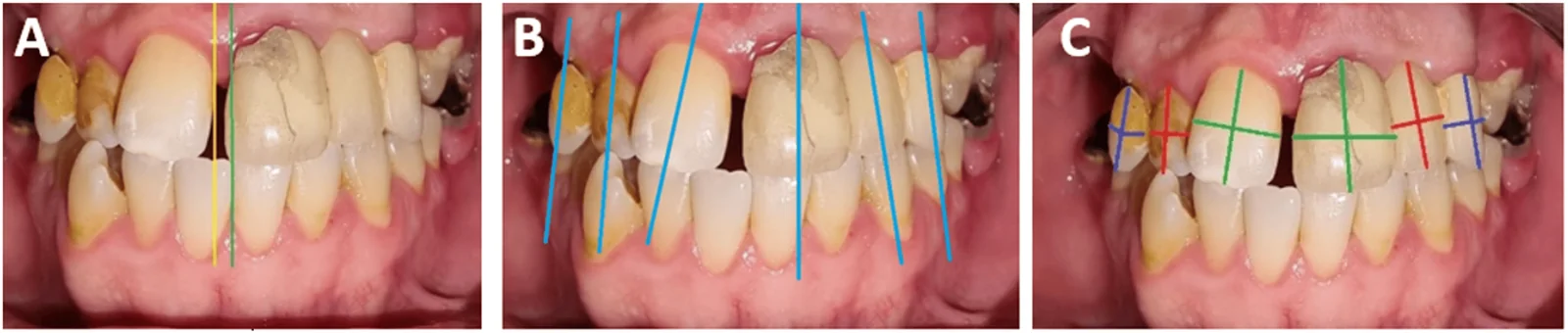
Aesthetic analysis showing the absence of alignment of median inter-incisal points (A), positional anomalies (B) and morphological anomalies (C) between the corresponding teeth

The aesthetic examination was performed with the existing bridge in place. The morphological, positional, and shade anomalies revealed were: a misalignment of the maxillary and mandibular inter-incisal points, associated with a significant inter-incisal diastema (Figure 3A), an asymmetric mesiodistal inclination of the anterior teeth associated with a loss of parallelism between the corresponding teeth, as well as a disharmony in dental alignment (Figure 3B). In addition, teeth 21 and 22 have larger mesiodistal dimensions than their corresponding teeth (Figure 3C), as well as a shade heterogeneity between the natural teeth and the prosthetic restoration.

Treatment decision

After explaining all possible therapeutic options to the patient, they refused the constraints of orthodontic appliances and potential orthognathic surgery. Therefore, a prosthetic solution was chosen to restore aesthetics and function. The decided treatment initially consisted of fabricating CAD/CAM-machined provisional tooth-supported fixed prostheses combined with a removable acrylic resin denture to replace the edentulous spaces. This was followed by conventional definitive fixed restorations (with a cast metal framework) combined with a cobalt-chromium removable partial denture (stellite type) to replace the edentulous spaces.

Clinical procedure

**Figure 4:**
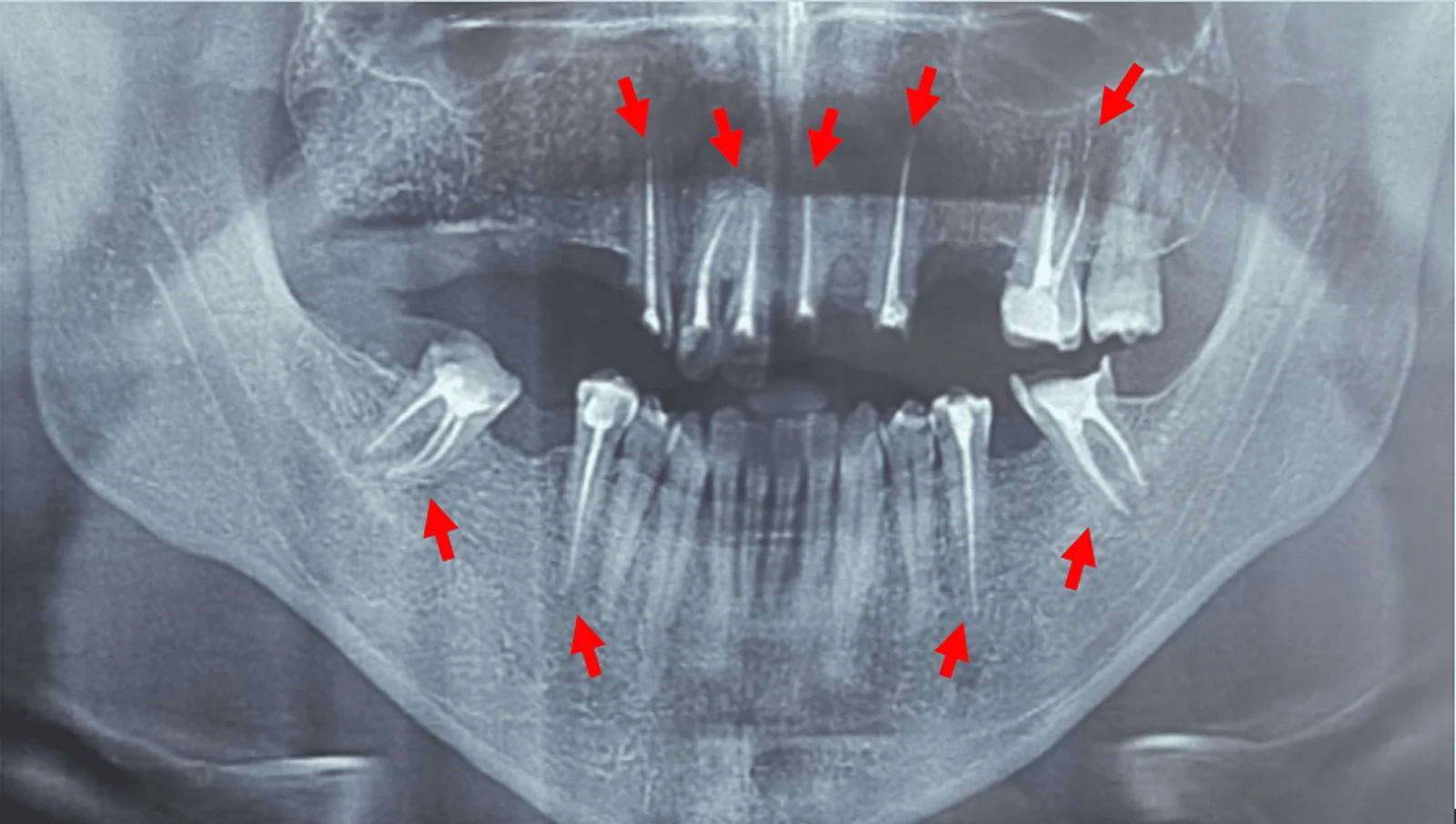
Panoramic radiograph; the red arrows show the future prosthetic abutment teeth treated endodontically

First, a cleaning phase was carried out by plaque control as well as supra- and subgingival scaling. Then, root canal treatments were performed on the abutment teeth of the future prostheses (Figure 4, red arrows).

Following stabilization of the dental and periodontal conditions by disappearance of signs of gingival inflammation (reddened gum with bleeding on probing), the digital pre-prosthetic planning phase was initiated.

**Figure 5:**
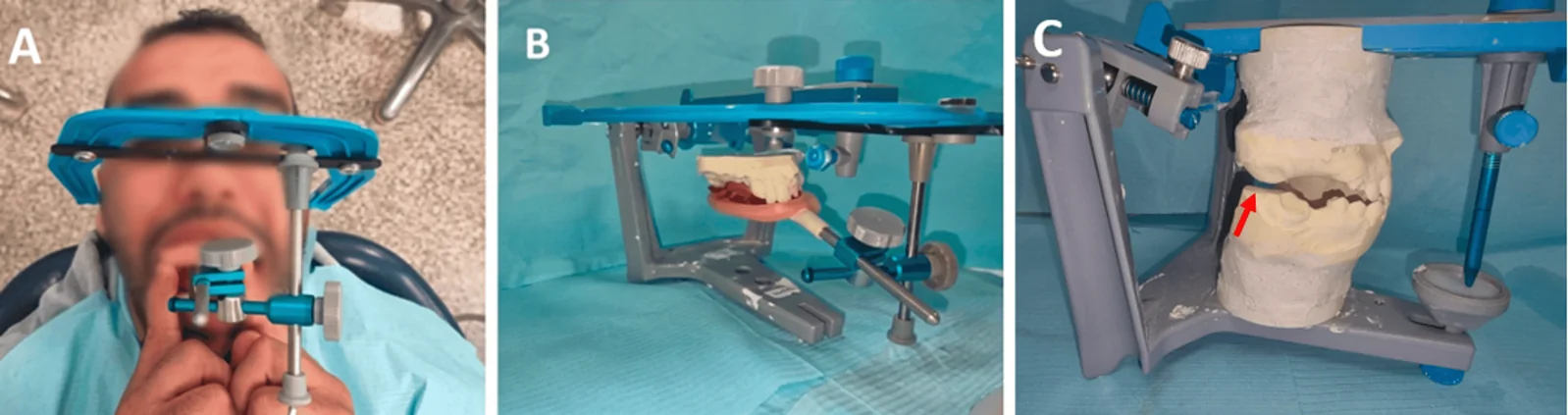
Recording the exact anatomical orientation of the maxillary arch using a facebow (A,B) and mounting the maxillary and mandibular arches on a physical articulator after elevation of the vertical dimension of occlusion (VDO) showing the increase in the occlusal-prosthetic space and the release of contact in the retro-molar areas (red arrow) (C)

Initially, the diagnostic models were mounted on a mechanical articulator. The maxillary model was transferred using a facebow (Figure 5A, 5B). Next, the vertical dimension of occlusion (VDO) was raised by 2 mm in the mouth to relieve contact in the retromolar areas and thus increase the significantly reduced prosthetic space in the posterior region to provide sufficient space for the insertion of future prosthetic restorations. For this, a resin bite registration block with a stent rim was used; the rim height was adjusted directly in the mouth until the desired elevation was achieved. The new vertical dimension of occlusion was validated using Willis's rule by comparing the nose-to-chin distance with a compass, which should be equal to the eye-to-mouth distance. In addition, the harmony of facial proportions, lip support, and smile dynamics were validated to avoid any risk of occlusal overbite.

Once the VDO was fixed, the lower rim was reduced by 1 mm to allow for the insertion of the recording material (ALUWAX; Aluwax Dental Products Company, Allendale, MI, USA) for the intermaxillary relationship. This recording was performed in centric relation (CR). To achieve this, we guided the patient's mandible by making small, gentle, upward rotational movements to obtain the highest possible position of the condyles in their glenoid fossa.

The articulator was programmed according to average values ​​(condylar slope at 30° and condylar angle at 0°), and the mandibular model was positioned in the maxillary model to the recorded position and then fixed to the articulator with quick-setting plaster (Figure 5C).

**Figure 6:**
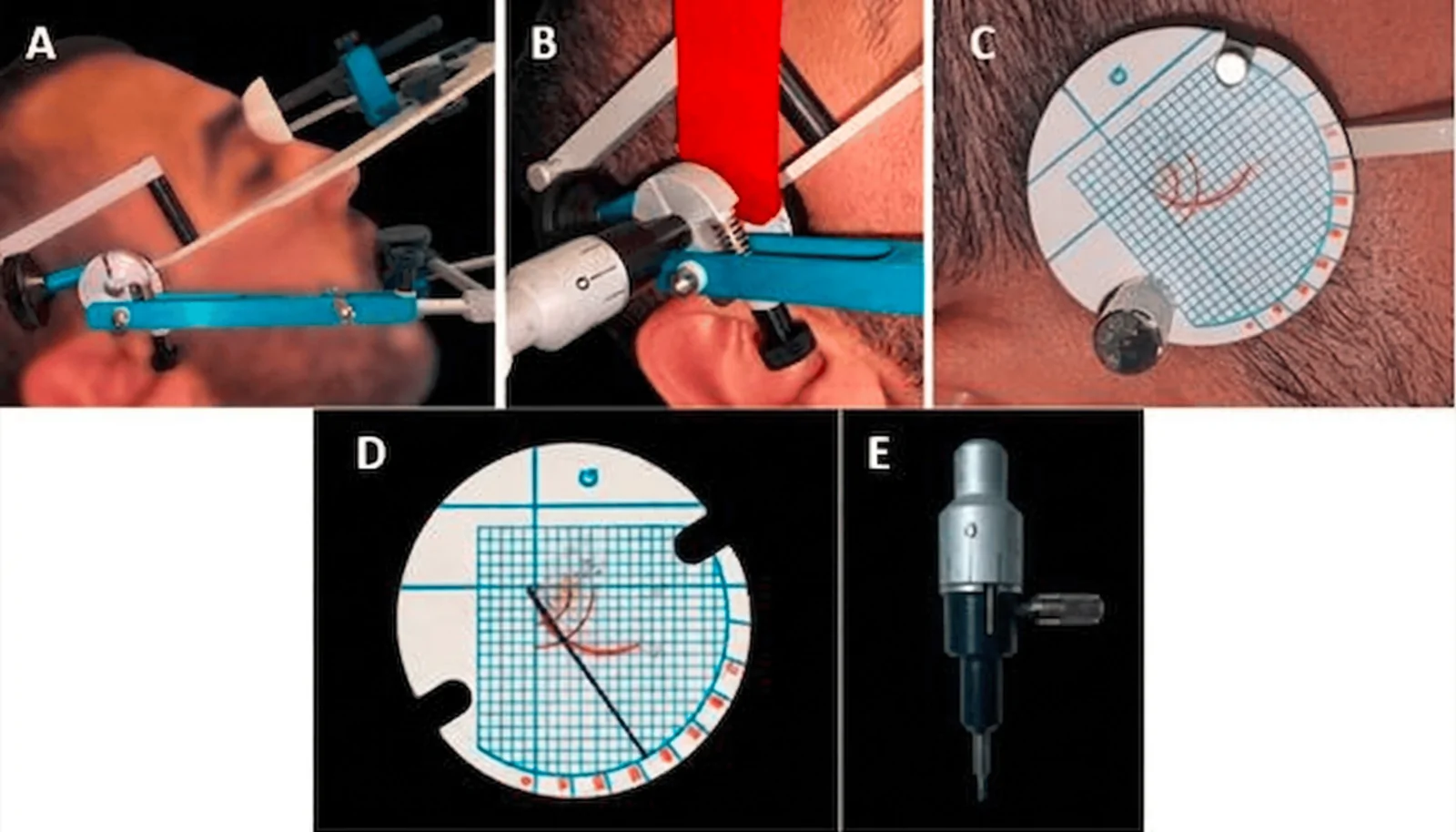
Recording of mandibular movements using the Quick Axis® mechanical axiograph (FAG Dentaire, Cergy, France). (A) Axiograph setup (B) A stylus is brought into contact with its lateral recording plate, then carbon paper is inserted between the two (C) The path traced by the stylus on the graph paper during the execution of the protrusion movement (D) The secant to the protrusion path determines a left condylar slope of 34° (E) The micrometer displays a left non-working lateral displacement of 0.6 mm, which corresponds to a Bennett angle of 10° according to the conversion table.

Mandibular movements were recorded using a Quick Axis® mechanical axiograph (FAG Dentaire, Cergy, France) (Figure 6A). Articular determinants were determined using a recording stylus positioned centrally against two lateral recording plates equipped with millimetric paper and placed adjacent to the temporomandibular joints.

A carbon paper sheet was inserted between the stylus and recording plate (Figure 6B). During protrusive movement, the stylus traced the protrusive pathway onto the millimetric paper (Figure 6C). The condylar inclination angle was determined by drawing a secant line through the protrusive pathway passing through the origin of the coordinate system and extending to the second arc of the recording field (Figure 6D).

During lateral excursions, a micrometer positioned at the end of the stylus measured the non-working side lateral displacement. The displacement value expressed in millimeters was converted into degrees using the manufacturer's conversion table (Figure 6E). After completion of the recording procedure on one side, the same protocol was repeated on the contralateral side.

To ensure that the measured values ​​​​were not distorted by handling errors, three successive tracings from three repeated recordings were merged into a single line (perfect superposition), which allowed us to validate the reproducibility of the movement.

**Figure 7:**
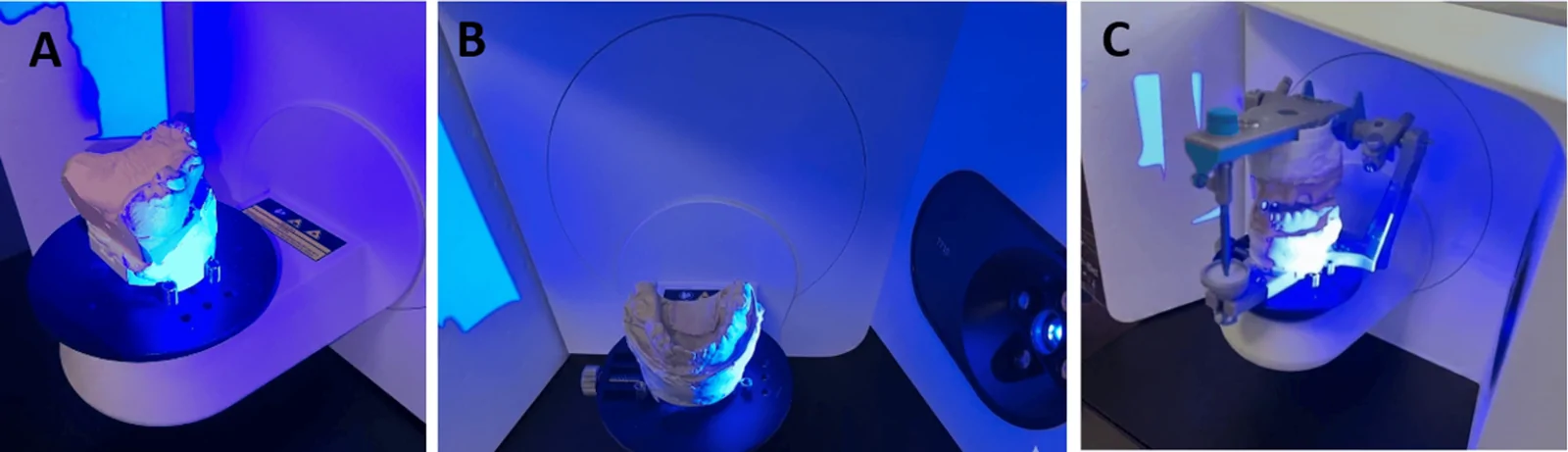
Digital acquisition of the maxillary cast (A), mandibular cast (B), and mounted casts (C).

Next, the physical articulator setup was transferred to the CAD software. This transfer was performed using a scanning table. Three digital scans were acquired: one of the maxillary arch (Figure 7A), one of the antagonist (Figure 7B), and finally, one of both arches in occlusion mounted on the physical articulator (Figure 7C).

**Figure 8:**
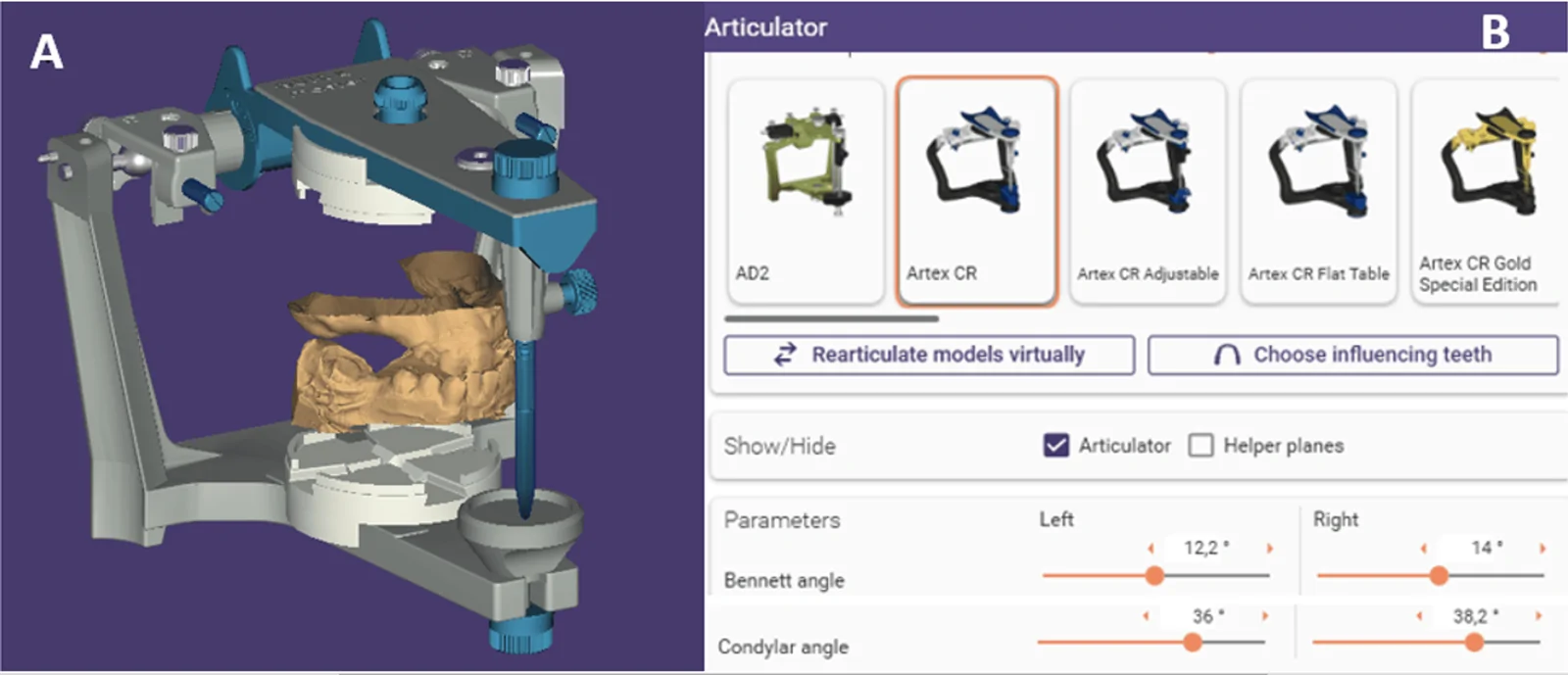
Captured photos of the DentalCAD ​​3.3 Chemnitz design software from exocad GmbH (Darmstadt, Germany): Virtual articulation of the diagnostic casts (A) and programming of the condylar housings according to individualized posterior determinants (B)

The DentalCAD ​​3.3 Chemnitz design software from exocad GmbH (Darmstadt, Germany) then allowed us to perform a three-dimensional comparison of the dental arches on a virtual articulator, the digital avatar of its mechanical counterpart, centrically linked to the new vertical dimension of occlusion (Figure 8A). It then offered us the possibility of adjusting the condylar brackets of this virtual articulator according to the actual values ​​of the condylar slope and Bennett angle obtained previously through mechanical axiographic recording, thus enabling personalized occlusal planning (Figure 8B).

**Figure 9:**
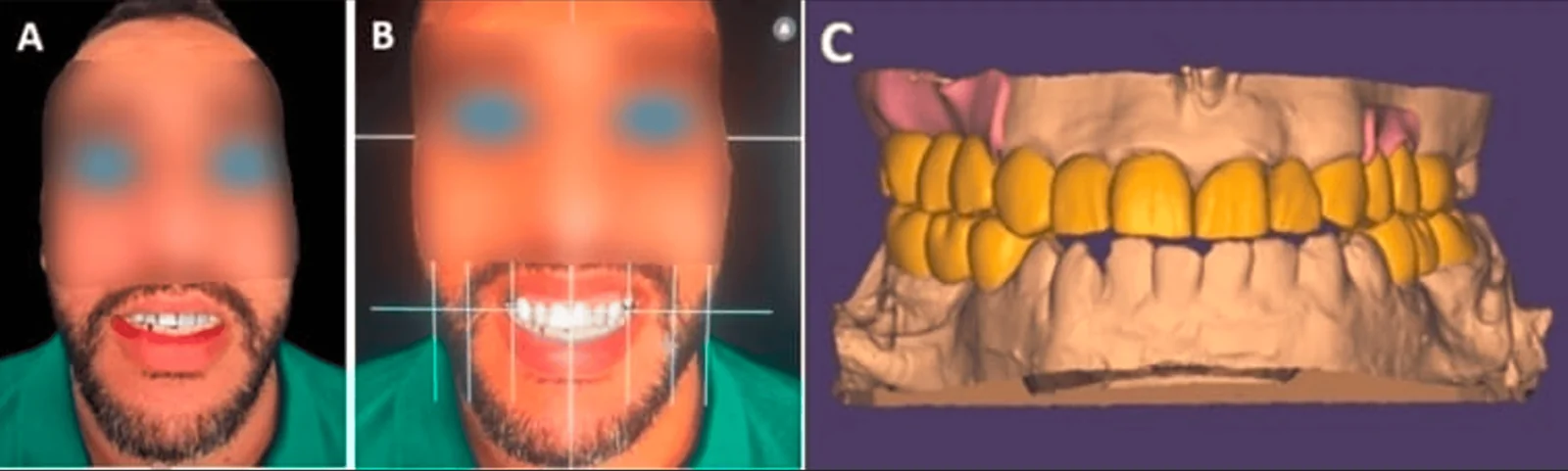
Digital integration of the future restorations within the patient's facial framework carried out by DentalCAD ​​​​3.3 Chemnitz design software from exocad GmbH (Darmstadt, Germany): (A) resting position; (B) smiling position; (C) restoration design without recreation of physiological overjet and overbite relationships.

Next, we moved on to the preliminary design of the future restorations, combining aesthetics and function. Aesthetically, the previously performed occlusal elevation had increased the vertical inter-arch distance in the anterior region, necessitating the fabrication of longer upper anterior teeth to cover the lower anterior teeth, which could negatively impact aesthetics. Furthermore, the mandibular advancement requires a greater labial tilt of the future restorations to ensure effective overjet. These two parameters guided us to find an aesthetic compromise using DentalCAD ​​3.3 Chemnitz design software without reproducing the physiological anterior inter-arch relationships. This was facilitated by the wide range of tooth shape and size choices offered by the software's digital library (Figure 9).

**Figure 10:**
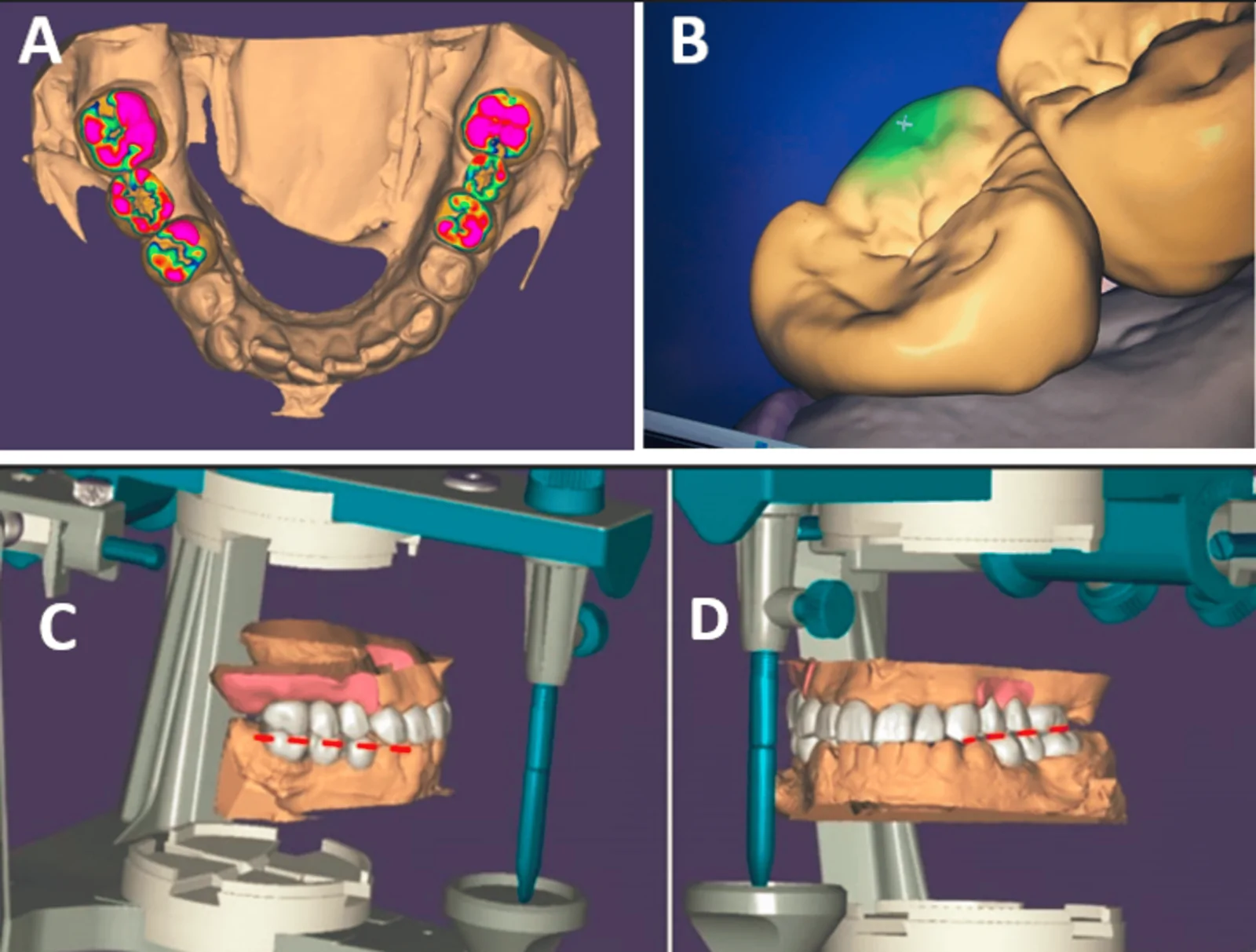
Captured photos of the DentalCAD ​​3.3 Chemnitz design software from exocad GmbH (Darmstadt, Germany): (A) CAD/CAM offers the possibility of precisely adjusting the occlusion by using colors to determine the occlusal clamping intensity for better static and dynamic equilibration. (B) CAD/CAM offers the possibility of modifying cusp height and slope. (C,D) Occlusal design featuring a nearly flat Curve of Spee, posterior group-function guidance, and absence of anterior contacts in maximum intercuspation. CAD/CAM: computer-aided design and computer-aided manufacturing

From a functional perspective, and thanks to the occlusal adjustment possibilities offered by the virtual articulator in static and dynamic occlusion (Figure 10A), an occlusal-prosthetic scheme meeting the patient's anatomical and functional requirements was established. At the posterior level, a preliminary design of the fixed and removable prosthetic restorations was created with reduced cusp height and slope (Figure 10B), tracing a nearly flat curve of Spee that allows for group guidance (provided by the posterior teeth) without risk of interference (Figure 10C, 10D). Contacts at the anterior level were eliminated in static occlusion to minimize vertical loads.

**Figure 11:**
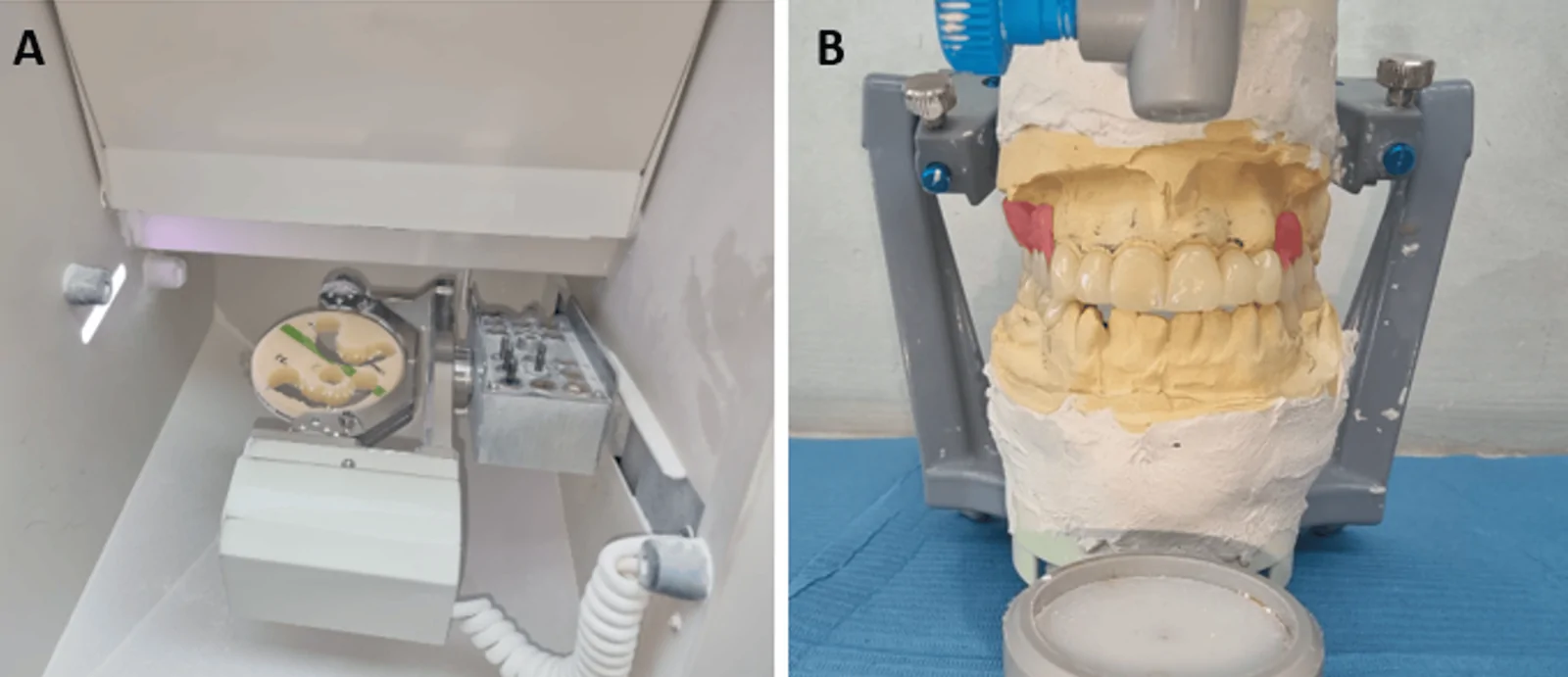
(A) CAD/CAM fabrication of provisional restorations in Ivoclar PMMA (Telio Lab, Ivoclar Vivadent AG, Schaan, Liechtenstein). (B) Physical realization of the virtual prosthetic project on a mechanical articulator. CAD/CAM: computer-aided design and computer-aided manufacturing, PMMA: polymethyl methacrylate

Once the virtual prosthetic design was digitally validated, the provisional restorations were fabricated. The fixed prostheses were computer-generated and milled from Ivoclar polymethyl methacrylate (PMMA) discs (Telio Lab, Ivoclar Vivadent AG, Schaan, Liechtenstein) (Figure 11A). However, the removable prosthesis was fabricated manually. The entire assembly was mounted on a physical articulator according to the occlusal concept chosen virtually (Figure 11B).

**Figure 12:**
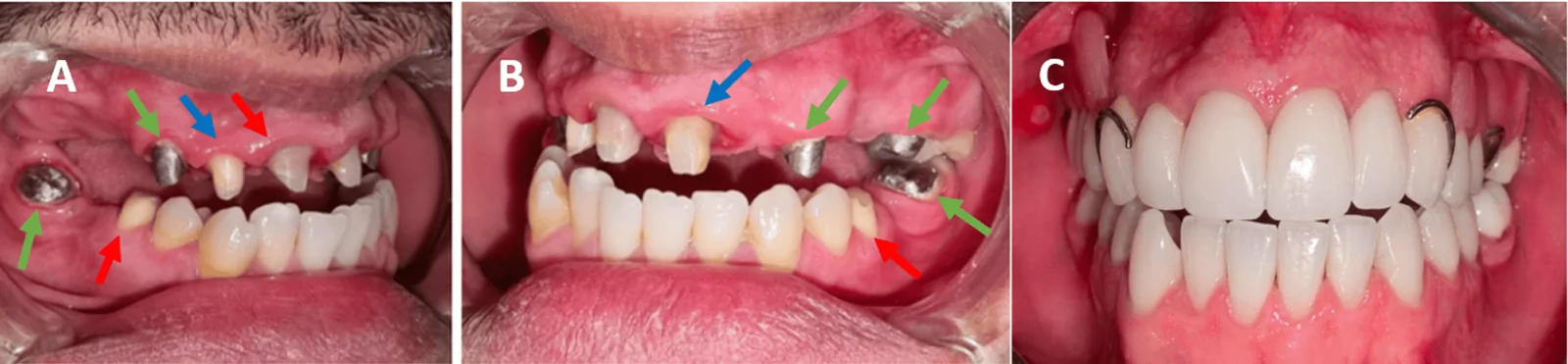
(A,B) Reconstruction of abutment teeth and tooth preparation. Red arrows: Teeth restored with direct composite. Blue arrows: Teeth restored with a bonded core build-up using a fiber post. Green arrows: Teeth restored with a metal inlay core. (C) Placement of provisional restorations.

Next, the abutment teeth were reinforced, depending on the degree of tissue loss. Teeth 11, 35, and 45 received direct composite restorations due to the persistence of four relatively high and wide walls after peripheral preparation (Figure 12A, 12B, red arrows). After preparing teeth 12 and 21, two remaining walls of sufficient height and width were retained, leading us to perform a bonded core build-up using a fiber post (Figure 12A, 12B, blue arrows). However, the extremely significant tissue loss after peripheral preparation on teeth 13, 23, 26, 37, and 47 led us to opt for a metal inlay core (Figure 12A, 12B, green arrows) before placement of the temporary restorations were used to maintain the new VDO (Figure 12C).

Occlusion was verified with the provisional restorations in place using articulating paper. In static occlusion, when the patient clenched their teeth, the articulating paper, placed between the dental arches, marked generalized contacts on the posterior teeth with no anterior contact. In dynamic occlusion, the interposition of the articulating paper between the dental arches during dynamic movements marked posterior guiding contacts with no anterior contacts during protrusion. However, during lateral movement, contacts were marked on the posterior teeth on the working side with no anterior or posterior contacts on the non-working side.

The provisional restorations were worn for three months. During follow-up appointments, the patient reported better adaptation to the new VDO with no discomfort or temporomandibular-related muscle and joint pain at rest or during speech and mastication.

**Figure 13:**
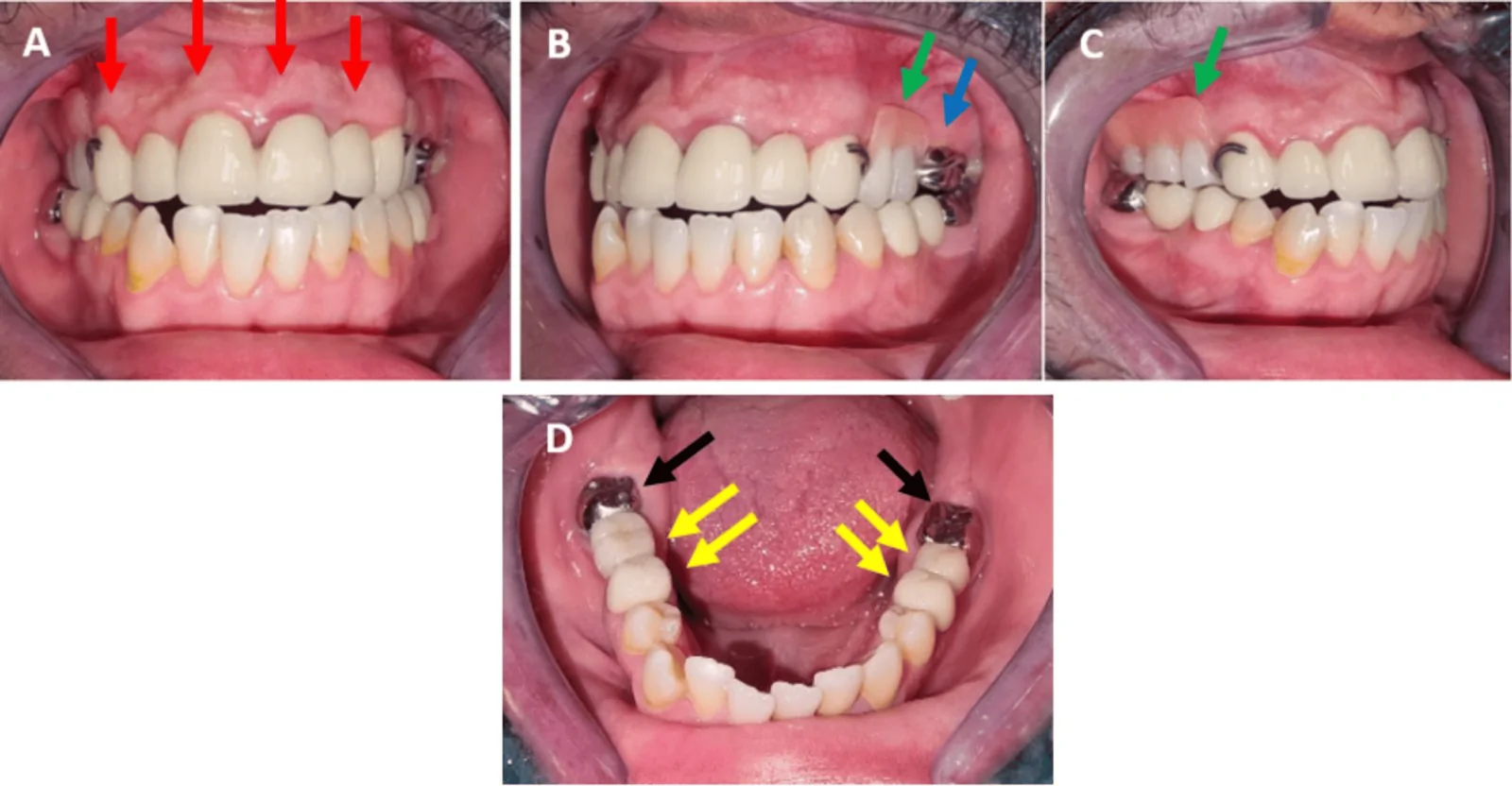
Clinical frontal (A), lateral right (B), left (C), and occlusal (D) views showing the final fixed and removable restorations. Red arrows: Anterior bridge from teeth 13 to 23. Blue arrow: Single crown on tooth 26. Green arrows: Removable partial denture (stellite type). Yellow and black arrows: The two posterior bridges replacing teeth 36 and 46.

Finally, the definitive restorations were put in place. In the maxilla, from teeth 13 to 23, a fixed bridge was chosen, fabricated with a cast metal framework onto which ceramic was mounted by manual layering (Figure 13A, red arrows). Tooth 26 was restored with a cast metal crown (Figure 13B, blue arrows). However, the maxillary edentulous area was compensated for with a cast cobalt-chromium alloy removable partial denture (Figure 13B, 13C, green arrows) integrated into the already chosen occlusal scheme. The decision to connect the anterior bridge from teeth 13 to 23 aimed to improve retention, given the significant stresses that will be exerted on the bridge during the placement and removal of the removable metal prosthesis.

In the mandible, the missing tooth 36 was replaced by a bridge supported by teeth 35 and 37, and the missing tooth 46 was replaced by a bridge supported by teeth 45 and 47. The units of bridges 35, 36, 45, and 47 were fabricated with a cast metal framework veneered with layered ceramic yellow arrows (Figure 13D, yellow arrows). However, units 37 and 47 were made with cast metal due to the tight occlusal-prosthetic space (Figure 13D, black arrows).

The validation of the final occlusion was performed using an articulated paper in the same manner already described with the provisional restorations.

The patient stated that he was satisfied with the aesthetic and functional outcome. No measurement scale was used to assess this satisfaction.

**Figure 14:**
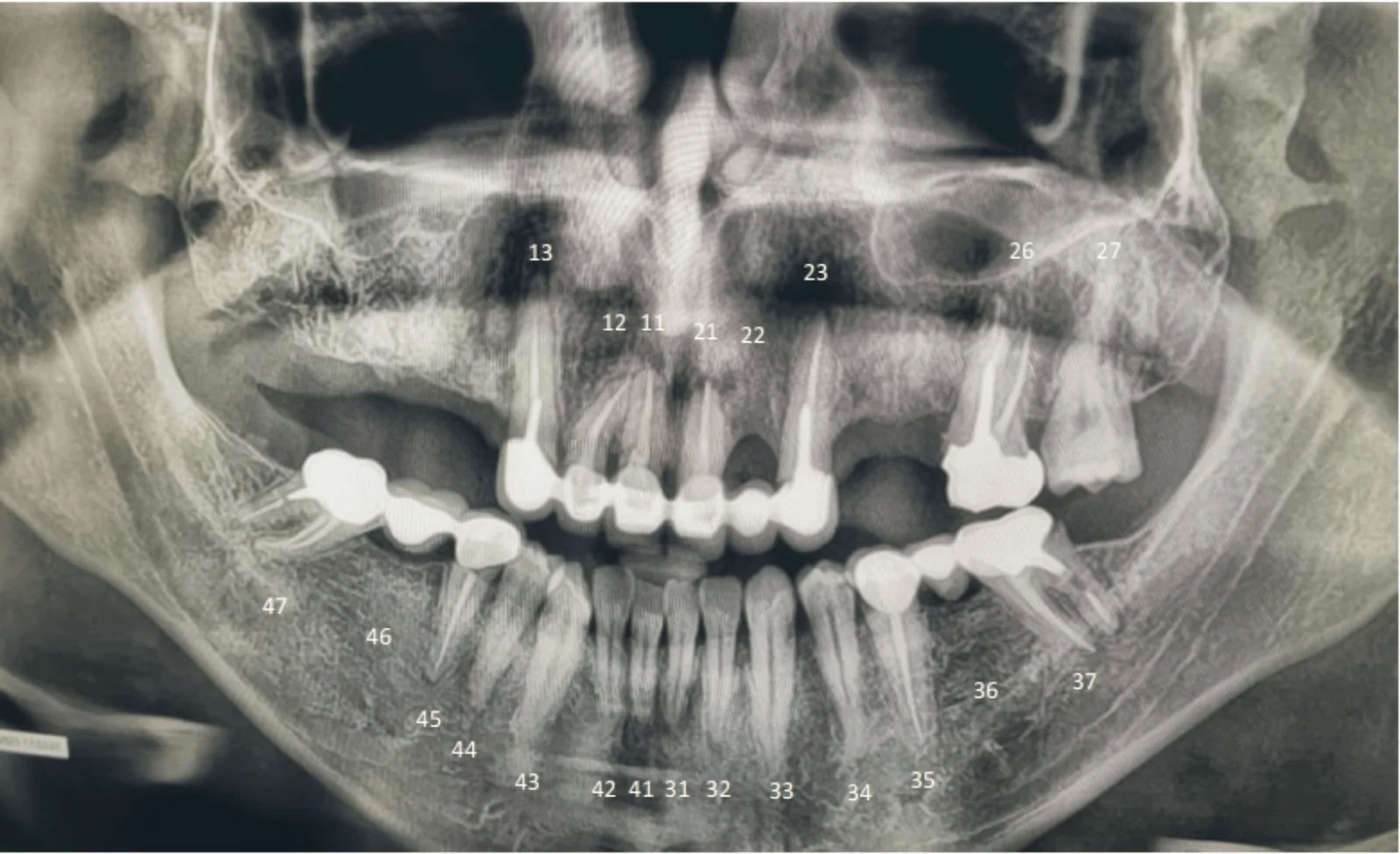
Panoramic radiograph taken after two years of follow-up with fixed prostheses showing the stability of the deep periodontium

After placement of the final restorations, the patient was examined after one week and then followed up every three months for two years. During this follow-up period, good occlusal stability was noted, and the patient adapted well to the new occlusal dimension. Oral hygiene was satisfactory, with no signs of gingival inflammation or deep periodontal involvement (Figure 14).

## Discussion

Restoring a non-functional anterior guidance with a fixed prosthesis is essential to ensure the longevity of the restorations and the balance of the masticatory system. A guidance is considered "non-functional" when it no longer fulfills its role of protecting the posterior teeth during eccentric movements (protrusion and lateral movements). However, when anatomical, skeletal, or periodontal constraints prevent the restoration of effective overbite and overjet, a therapeutic compromise will be sought, aiming to minimize these constraints by establishing a well-adapted biomechanical approach while maximizing visual harmony.

The occlusal-prosthetic concept chosen to manage the impossibility of restoring the physiological guidance of our patient is as follows: No contact between the anterior teeth in maximum intercuspal position (MIP) to minimize shear stress. Absolute posterior stability; the posterior teeth must have simultaneous, generalized, and well-distributed contacts with no premature contact in MIP, as well as guidance ensured by a group function; all the posterior teeth must participate in the guidance to minimize mechanical stress.

In our case, CAD/CAM planning validated the selected occlusal scheme and the provisional restorations allowed for clinical evaluation and confirmation of the chosen concept. They also enabled assessment of the increase in vertical dimension before the fabrication of the final prosthesis.

Indeed, Aldowish et al. in 2024 confirmed in their literature review that if anterior guidance cannot be restored, group function combined with a uniform distribution of forces may represent one clinically acceptable compromise. Furthermore, they concluded that the absence of overjet and overbite is no longer considered a pathological clinical situation provided that posterior support is perfect [9]. However, Chen et al. in 2023 conducted a clinical study to evaluate muscle activity during different occlusal schemes using electromyography. They concluded that in patients with pre-existing temporomandibular joint disorders, group function without functional guidance can delay muscle relaxation [10].

In this case, a mathematical virtual articulator (simulates jaw movements using trigonometric calculations) [11] integrated into the CAD software was programmed with the recorded values ​​of condylar inclination and Bennett angle. Prior to digitalization, casts were mounted on a conventional mechanical articulator and subsequently scanned to obtain a virtual replica of the clinical situation. This dual mounting protocol was selected because the patient lacked both a physiological occlusal plane and functional anterior guidance. Under such circumstances, relying exclusively on a standard virtual mounting procedure would have introduced a significant risk of error. The use of a facebow transfer proved indispensable for accurately determining the spatial position of the maxilla relative to the cranial base. Furthermore, the presence of extensive edentulous areas justified recording the intermaxillary relationship in centric relation, thereby ensuring a reproducible and non-arbitrary positioning of the dental arches. This dual physical-digital workflow remains a useful clinical procedure that simulates mandibular movements based on the patient's actual anatomical values.

Martani in 2024 reported that dual mounting secures the digital workflow through mechanical validation. Using the physical articulator as a reference before scanning ensures that the vertical dimension recorded in the software is strictly identical to that validated clinically [12]. However, there are other types of fully adjustable articulators, such as the MODJAW system (Tech in Motion, Villeurbanne, France), considered the most accurate for reproducing intermaxillary relationships (IMR) because they eliminate errors related to handling plaster models, allowing real-time capture of mandibular movements using ultrasonic transmitters and receivers. According to an in vitro study by Nagy et al., the MODJAW system demonstrated an accuracy of 9.7 ± 1.76 µm and a trueness of 10.8 ± 1.40 µm for recording static IMRs [13]. Furthermore, King et al., in an in vitro study, also showed that MODJAW allows for much finer reproduction of the patient's actual dynamic trajectories than physical articulators [14].

In our case, the aesthetic compromise, within the patient's antomo-functional context, was guided by the valuable options offered by the design software. This software boasts a comprehensive library of dental shapes, which allowed us a very wide range of choices through intelligent, computer-aided morphological suggestions tailored to the clinical situation. Furthermore, it offered the possibility of automatically tracing facial reference lines and integrating patient photographs to align the 3D models with facial features. The results obtained were transferred to provisional restorations, enabling validation of the aesthetic design and communication with the patient.

In the same regard, design software used with fully adaptable articulators allows the creation of a true 4D avatar of the patient by combining intraoral scans (teeth), facial scan (face) and dynamic recording of mandibular movements allowing the smile to be integrated into facial dynamics [15].

However, despite the increasing development of digital systems, conventional methods still retain a slight advantage in terms of aesthetic detail. Pascal Magne et al. concluded in 2002 that the layering of ceramics by hand sculpting allows for the creation of more random and therefore more natural micro-textures (Hunter-Schreger striations, growth lobes) than surfaces generated by computer-aided manufacturing [16].

Indeed, the aesthetic outcome of conventional ceramic-metal restorations in this clinical case demonstrates the clinical feasibility of the synergy between the precision of digital workflow algorithms which enabled the clinical validation of the planned occlusal compromise and the craftsmanship of the prosthetist for better management of our clinical cases.

## Conclusions

Managing a non-functional anterior guidance system that cannot be restored with a fixed prosthesis requires the practitioner to adopt a reasoned therapeutic compromise approach, dictated by the patient's anatomical and skeletal constraints. In the case presented, the impossibility of recreating a physiological overjet and overbite, imposed by the Class III mandibular prognathism, led to the choice of an occlusal-prosthetic concept based on eliminating anterior contacts in static occlusion and establishing posterior group guidance, in accordance with current literature.

The contribution of CAD/CAM has provided us with the precision of the intermaxillary transfer using a double mount, the dynamic simulation of occlusion with a mathematical virtual articulator, and the design of a facially integrated prosthetic project all provided tools for objectively assessing and validating the chosen compromise before any final fabrication.

This case illustrates that, despite the limitations of the anatomical substrate, digital planning remains a feasible therapeutic option for managing a Skeletal Class III patient. The anticipated developments in dynamic capture systems and 4D facial avatars offer promising prospects for even greater individualization of these complex clinical situations.
